# Health data sovereignty: pinpointing conceptual ambiguities

**DOI:** 10.3389/fdgth.2026.1820189

**Published:** 2026-08-24

**Authors:** Cristian Timmermann, Javiera Atenas, Bianca Jansky, Wannes Van Hoof, Frank Ursin

**Affiliations:** 1 Institute for Ethics and History of Health in Society, Medical Faculty, University of Augsburg, Augsburg, Germany; 2 School of Business, Arts, Social Sciences and Technology, University of Suffolk, Ipswich, United Kingdom; 3 Section for Health Service Research, University of Copenhagen, Copenhagen, Denmark; 4 Data Governance Directorate, Sciensano, Brussels, Belgium; 5 Institute for Ethics, History and Philosophy of Medicine, Hannover Medical School, Hannover, Germany

**Keywords:** digital bioethics, digital infrastructure, governance, public goods, self-determination, solidarity

## Abstract

Collection of health data has increasingly become central within healthcare and public health, and various actors such as clinicians, researchers, policymakers and patients are increasingly voicing claims to being able to responsibly control data. In this context, the concept of health data sovereignty (HDS) has had highs and lows in bioethics and policy discussions over the years without being either fully discarded or established. We argue that while there is a persistent discussion on defining the underlying concepts of “health” and “data”, the real hurdle in the widescale adoption of the concept of HDS in research and policy lies in substantial disagreements on the scope and implications of the concept of sovereignty. We argue that before the concept of HDS can have a meaningful impact, we need to clarify five issues: (1) lack of consensus over who should claim sovereignty, (2) the public goods problem of sovereignty, (3) reliably maintaining sovereignty, (4) the need to protect the reasonable interests of all stakeholders, and (5) the links between solidarity and sovereignty. In mapping these conceptual challenges and the tensions between them, we hope to ignite new policy efforts and community-level grassroots initiatives towards HDS based on ethical deliberations.

## 1 Introduction

In contemporary digitised and data-driven healthcare systems, the question of who “owns” health data has shifted from an abstract concern to a concrete, real-world issue. Traditionally, health data were primarily used for clinical purposes and stored within proprietary local systems. However, understandings of what health data can be used for, what value they generate, and for whom, are increasingly shifting and becoming blurred. At the same time, the production of health data is no longer confined to clinical settings, as individuals generate health-related data through the use of commercial tracking devices (1). This is accentuated by the fact that health and healthcare are increasingly shaped by ‘health data gathering’ Big Tech offering wellness commodities that blur the boundaries between medicine and every-day care resulting in both concepts becoming fluid (or liquid) and merging (2). In addition, Big Tech is entering the public health and healthcare sphere through services and infrastructures that grant them increasing power and control over health data (3).

Ethical deliberation regarding the implementation of the concept of data sovereignty in the health sector is however stalled. While supporters offer analyses justifying its inherent values (4, 5), others break the concept down to advance its constituent components (6) – until it no longer can have its intended impact. In effect, neither clinicians, researchers nor patients have conceptual clarity, which is needed to demand from policymakers the level of control they expect to retain over health data. Without an agreement over concepts, needs cannot be articulated. Therefore, more clarity is needed on what raises controversy over the concept of health data sovereignty (HDS) and what stakeholders expect it to deliver. This conceptual clarification seems particularly relevant in light of current attempts to regulate health data, as the European Health Data Space (EHDS) (7) exemplifies. Conceptual clarity can unleash the potential the concept of sovereignty has to unite different stakeholders to overcome their differences, recognise the power that they can gain through collective action, and create awareness about the actions required to have control over health data decisions.

To ensure that HDS can meaningfully inform policy development it is necessary to reduce ambiguities for stakeholders and policymakers (8). This then also prevents the risk of a concept being co-opted or hijacked. Conceptual co-opting refers to a process in which powerful stakeholders misuse politically significant terminology to advance their own agenda (9). In this article, we argue that a major problem with the concept of HDS is that while all three underlying concepts – health, data, and sovereignty – are subject to ambiguous specifications by governments, institutions, and academia, the core challenges and tensions lie in the third element: “sovereignty”. We aim to disambiguate sovereignty by explicating its relational dimensions towards health data. This should not be interpreted as downplaying the complexity of the concepts “health” and “data”. For instance, “health” has a very broad spectrum of definitions ranging from “absence of disease” to the “highest attainable standard of physical and mental wellbeing” (10). Similarly, while there are some widely accepted definitions of data, it is hardly possible to specify which data are relevant for people in relation to their health status now or in the future (11). Health data should not be understood as an end in itself, but as the foundational layer within the data–information–knowledge–wisdom hierarchy (12): it serves epistemic goals in healthcare, public health or clinical research, which underpins its relevance for practical decision-making.

Despite the difficulties in agreeing on what constitutes “health data”, the imperative to protect privacy, avoid harm, limit discrimination, and respect autonomy has built sufficient consensus to overcome differences in terms of conceptual interpretations to work together and pursue a common agenda [see e.g., the definition of health data in the General Data Protection Regulation (GDPR) (13) recital 35]. In terms of its conceptual history, sovereignty, unlike health and data, was not subject to bioethical discourses but has a long tradition in political philosophy and theory (14, 15). Here we can find both: advocates for governmental control, particularly through a head of state, and those defending rights of self-determination for citizens. It is important to keep these conceptual developments in mind when assessing the application of the concept of sovereignty over health data and its different interpretations in health data policy and practice.

So far bioethical discussions of the conceptual ambiguity of health data sovereignty remain scarce. The most significant contribution is Hummel et al.'s systematic review (6), mapping the conceptual landscape of data sovereignty across 341 publications, identifying its descriptive variability across agents, contexts, and values. Our contribution differs in two respects. First, we narrow the scope to health data, where the stakes of sovereignty differ from generic data contexts due to e.g., embodiment, different interests in privacy among genetically related persons and groups, and its common good character. Second, where Hummel et al. offer a descriptive taxonomy, we develop a normative account: we identify five dimensions that any legitimate exercise of HDS should aim to address, and we justify these dimensions through wide reflective equilibrium (see Methods). We stipulate that the main hurdle in the wider adoption of the concept of “health data sovereignty” lies in its third element: the widely differing interpretations and values attached to being a sovereign. We therefore proceed by examining the different interpretations and values that come into play when appealing to sovereignty.

## 2 Methods

This study employs conceptual-ethical analysis to examine the conceptual foundations and ethical implications of different conceptions of sovereignty in HDS. The objective is not to provide empirical measurement or descriptive mapping of stakeholder positions, but to clarify conceptual ambiguities. For methodological transparency, we will explicitly outline our analytic procedure. The methodological core of this study is a conceptual-ethical analysis of HDS (16). We identify sovereignty as the principal source of normative disagreement and practical uncertainty.

The analysis proceeds in three steps. First, we examine the historical and philosophical foundations of sovereignty by a conceptual exploration, drawing on political philosophy [specifically on participation (17–19)], public goods and commons theory (20, 21), and data and digital governance literature (22–24). This step identifies competing interpretations of sovereignty (e.g., individual, collective, state-centred) and their ethical implications (5, 6). To this end, we conducted non-systematic literature searches, identifying relevant sources through targeted database searches, citation tracking via backward snowballing, and recommendations from colleagues across the represented disciplines. Second, we differentiate HDS from adjacent normative constructs such as privacy, informed consent, ownership, and digital sovereignty. This allows us to clarify whether HDS adds distinctive normative value or merely rearticulates existing principles. Third, based on this exploration and delineation, we develop an account of HDS intended to guide health data governance by a normative reconstruction. By identifying ambiguities and normative tensions, we aim to prevent the instrumentalisation of the concept of sovereignty by dominant actors (“conceptual capture”) and to provide criteria for ethically robust, pluralist, and democratically legitimate health data governance.

Our conceptual-ethical analysis was informed by the method of wide reflective equilibrium, i.e., justifying our account by building coherence between moral judgments, ethical principles and theories (25). In practice, this meant that initially considered moral judgments towards sovereignty in HDS were discussed within the author team. Given the interdisciplinary composition of the author team (bioethics, political philosophy, data governance, information science, science and technology studies, and public health), disagreement was a productive resource. The analytic process proceeded iteratively: each author drafted contributions reflecting the considered judgments and theoretical commitments of their disciplinary tradition, and successive rounds of internal review and discussion surfaced tensions between these contributions. We resolved disagreements by returning to the underlying ethical principles at stake, testing competing formulations against paradigm cases, and revising both judgments and principles until the team reached consensus. This procedure approximates wide reflective equilibrium at the level of the author team rather than the individual analyst.

Hence, we integrate established bioethical principles (e.g., autonomy, justice, solidarity) with political-philosophical accounts of sovereignty and collective self-determination. We explicitly address power asymmetries, collective-action problems, and risks of majoritarian domination. The concept of HDS was examined not only as a claim to control, but as a governance arrangement requiring ongoing maintenance, participation, and institutional design. To systematically address the principal areas of disagreement, we organised our analysis into five thematic clusters derived from iterative engagement with academic literature, policy documents, team discussions, and identified conceptual tensions. This thematic structuring allows complex normative issues to be analytically disentangled and evaluated in a structured manner.

## 3 Results

**Figure 1:**
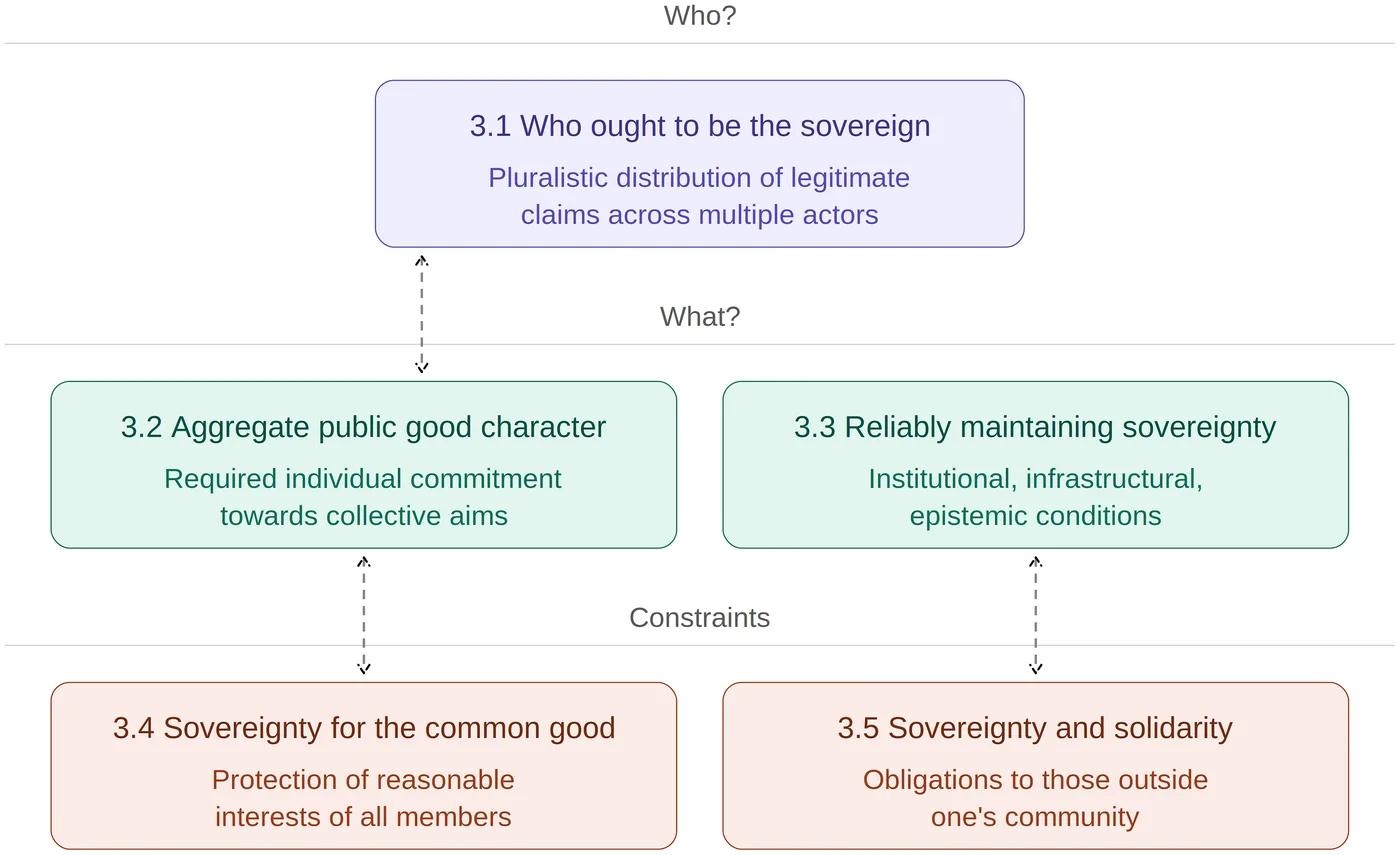
Structural account of the five dimensions identified in our analysis (sections 3.1–3.5) organised into three functional tiers: who may claim sovereignty, what it requires, and the constraints under which it remains legitimate. Double-headed arrows mark the three tensions identified in section 3.6 (Source: developed with Claude Opus 4.7 based on section 3.6 and further revision by the authors).

The following section presents the results of our conceptual-ethical analysis. These results take the form of establishing the normative dimensions of HDS based on ethical arguments and reflective deliberation rather than empirical findings. We proceed by showing that the real hurdle in the wider adoption of the concept of HDS lies in the widely differing interpretations and values attached to being sovereign. For this purpose, five thematic issues are examined over which greater consensus and awareness need to be reached: (3.1) lack of consensus over who should claim sovereignty, (3.2) the public goods problems of sovereignty, (3.3) meaningfully maintaining sovereignty, (3.4) the protection of reasonable interests of all stakeholders, and (3.5) the links between solidarity and sovereignty (see Figure 1).

### 3.1 Who ought to be the sovereign over health data?

Perhaps the most essential problem with HDS is that there is no consensus on the normative question *who should hold sovereignty and what its scope should be* in relation to health data. This involves a major risk of conceptual capturing, when those in or with power claim to be representing people's voice and interests (6, 26). In some countries, for instance, heads of states have claimed to be speaking on behalf of the people and end up equating sovereignty with governmental control (27).

There are, however, vast differences between having the power to impose the will of individuals, a political party, or industry on others, and exercising sovereign rights as a collective of people. Particularly in these extremely unequal times, those in power may show epistemic ignorance regarding the interests and needs of those over whom they exert sovereign power, specifically when legislating about the needs of those in lower socioeconomic, marginalised or vulnerable positions (28). In increasingly heterogeneous societies, it is also likely that the interests and concerns over how health data on members of ethnic and racialised minorities is collected, analysed and used, are not given adequate consideration (26, 29–31). Therefore, those affected may not understand themselves as data sovereigns, as Indigenous scholars have repeatedly stated (5). Moreover, feminist scholars have documented how patriarchal structures in health and science policy may still grant power to some groups to define whose health data matters and for whose health needs data are collected, often disadvantaging women and girls (23, 32, 33). Similarly, intergenerational justice scholars have shown how the interests of younger generations are routinely sacrificed and there is a tendency to prioritise short-term interests at the cost of long-term planning (34). If sovereignty is to be achieved through representation, either representative governments or spokespersons, it is crucial that those taking decisions on other people's behalf have solid knowledge and evidence of their needs and are acting in their interests. A pluralistic framework is required where no single authority holds absolute power over determining the needs and objectives that enhance the well-being of the governed (35).

Individuals and communities may have strong interests in having agency and being sovereign over health data relating to them. They may claim ownership as health data is essentially derived from their status as persons and people cannot fully dissociate from their data (36). They may also claim that they need control rights over health data, due to interests in privacy and to be able to establish measures to reduce possible harms (37). In some cases, individuals may demand shared control over health data, as individual vigilance may not suffice (38) and decisions by others over their own data may lead to certain forms of group harms (39). In such cases, in contrast to many other social institutions and systems, individuals cannot migrate to other jurisdictions whose rules are more compatible with their interests, as data crosses borders and may reveal sensitive health information irrespectively of where data were collected and shared. Furthermore, even when people have options to refuse further health data processing, these often come with minor or major disadvantages (40). Refusals to share data may also need to be recorded and may thus generate additional data (41). These disadvantages are, however, not equitably distributed among the population and are likely to disadvantage groups who are already in a less favoured position, such as homeless, ethnic and racialised minorities. For such reasons, it is more effective to join others to influence data policies instead of merely exercising alternatives to opt out or attempting to migrate to jurisdictions which are more favourable to one's needs and interests. In the context of the EHDS, the opt-out approach has been criticised for bringing in the illusion of individual control, when in fact the opposite is true due to the limited impact of a single opt-out and the introduction of many exceptions to this right (42).

At the same time, individuals and groups claiming sovereignty in terms of having complete ownership of health data need to recognise that a wide range of individuals and institutions are needed to create and maintain health data. Several actors can therefore have justifiable claims to health data: individuals, their genetic relatives, health workers and technicians involved in creating the data, institutions that store, process and utilise data as well as the states which provide the needed infrastructure (43). Individuals and genetic relatives may claim control rights over health data to protect fundamental interests such as privacy. Health workers, data scientists and governments can also claim control over health data to reward their investment and added value for such a resource (44). Furthermore, institutions and industry that have collected and curated health data following ethical and legal guidelines may have the right to sell and buy data over which they have legal ownership titles. Researchers and technology developers are a group with special interests and claims, as they generate much of the context and tools for health data to gather value. Liberal political traditions recognise rights to benefit from and have a say over things one has mixed labour with, such as technological innovations (45).

### 3.2 Acknowledging the aggregate public goods character of sovereignty

Sovereignty has characteristics of *aggregate public goods* with well-known problems associated with this type of goods (46). That is, when a group of people with compatible interests works towards establishing sovereignty, many others can enjoy the benefits of a system that protects their interests in relation to health data without contributing to its existence and maintenance. Lack of participation in collectively establishing and defending sovereignty weakens the capacity to control health data.

To counter this problem, there needs to be a greater awareness among those desiring sovereignty about the fact that exerting sovereignty over a good implies being active. Sovereignty needs to be built, defended and maintained, and therefore sovereignty comes as much with responsibilities as it does with rights to control (47). Maintaining sovereignty involves much more work than passive possession.

The fact that people need to cooperate to establish sovereignty has advantages. When individuals with similar interests and needs are brought together, they can unite a considerable mass of people with a wide range of skills, knowledge and experiences to counterbalance institutions with competing interests. In times when Big Tech have concentrated large enough economic and political power to compete with smaller countries, communities seeking sovereignty need to attract a massive number of members to build a critical mass that is able to effectively act and defend itself as a collective (48). This is something that can only be achieved by getting familiar with the interests of others, harmonising demands, overcoming differences and cooperating to advance common interests (49). Furthermore, all stakeholders need to be aware of the value of data ecosystems and the risks of eroding trust for collecting, processing and utilising data (50). In addition, there needs to be greater awareness that solely relying on individual informed consent processes to remain cognisant of health data uses, risks and opportunities may be insufficient to defend one's interests in today's world of big data (51). To ensure access to a more complete range of relevant information about data handling and to demand that credible efforts are made to keep such information understandable for non-experts may require collective action to exert pressure on industry and policymakers. Therefore, a wider understanding of HDS and aggregate public goods requires recognition of the importance of continuously maintaining trustworthiness and seeking ways to discourage free-riding. Maintaining sovereignty requires constant watching that good data practices are kept.

A special challenge for HDS is that companies can gather massive amounts of information about whole communities and populations when certain individuals deviate from collective efforts by offering their personal information. Individual choices (or vulnerabilities in the case of theft) can have major repercussions on the privacy of groups (52, 53). This is a major issue when genetic data from a few individuals can be used to track down other members of the same community or give access to group-specific health status information. For example, a report by FairPlanet discusses how genetic data sourced from Indigenous territories can inadvertently expose group-specific traits, such as health vulnerabilities or predispositions, even if only a few individuals’ DNA is analysed. This can lead to exploitation or misuse, as corporations or states might use such insights without proper consent or benefit-sharing agreements, potentially harming the entire community's interests (54). It is a particularly troubling issue that others who are biologically related or suffer from a similar genetic condition are usually still able to share health data which may end up interfering with the interests of those who oppose the diffusion of health data because of reasonable concerns. We already can observe the use of genetic data by police, military and security forces to identify and track minority groups, undocumented migrants and activists (55). While these uses can have positive aspects, such as the identification of unknown decedents in US-Mexican borderlands (56), they come with substantial risks when, for instance, mass collection of genetic data in China can allow discriminatory profiling of related family members (57).

A major fairness issue arises when communities attempt to retain sovereign control: individuals who opt to sell their personal data to maximise their individual interests are likely to sell their data at higher price when deviating from the majority. Especially where the majority chooses to refuse to give away data in order to advance common goals such as maintaining privacy and reducing risks. Consequently, achieving sovereignty requires a certain level of coercion to respect rules agreed by a majority. This is a shared characteristic with most common-pool resources (58). One is obliged to either question full personal ownership of health data and thereby defend the position that individuals do not have the right to dispose of health data as they see fit (43), or admit that some people will be compelled to participate in a community by following their rules, and thereby accept a certain form of benevolent paternalism over health data decisions (59). Regarding the latter position, we need to remember that people usually surrender rights to be part of a community, to advance their most valuable interests and enjoy the advantages of peaceful social coexistence (60). This may oblige people to transfer some informed consent requirements regarding data from the individual to the group and do their share in ensuring health data uses are adequately monitored to achieve health data sovereignty as a community (61).

### 3.3 Difficulties in reliably maintaining sovereignty

Sovereignty needs to be *reliably maintained*. People must maintain the willingness and capacity for self-determination to control their health data. This requires developing and effectively using skills and knowledge to exert self-determination and defend the right and ability to do so. By definition, people cannot alienate sovereign rights while claiming to be sovereign.^1^ Large power asymmetries between data users and data subjects (38), particularly in the context where health data is exchanged or commodified, require substantial active participation to defend sovereign rights (63).

To maintain sovereignty, it is not enough to effectively shield health data from unwanted or irresponsible uses, but also to make rational use of this resource. Here it is important to not understand data as something static: health data “travel beyond their place of production” (64) and therefore have a certain life of their own. They are constantly generated and regenerated, potentially impacting people's lives, and can have commercial and scientific value (65) beyond the person initially connected to the data (66). Therefore, it requires continuous engagement to explore the potential uses of health data that ought to be supported and those to be prevented. For health data that is of general interest to be shared, there needs to be some type of monitoring over how data ends up being used, and it would be a task in which those striving for sovereignty would have to get involved. As monitoring involves work, a level of sovereignty that can be reliably maintained needs to be identified, to reach a balance between the control some citizen's demand and not overburdening other citizens with excessive control tasks. Civic monitoring strengthens collective data sovereignty by enabling citizens and communities to generate, steward, and interpret shared evidence. It plays a vital role in scrutinising government actions, to promote transparency and accountability, while also fostering spaces for deliberation and discussion of policy alternatives. Furthermore, civic monitoring supports active participation and collaboration in the design, implementation, and evaluation of public services, ensuring that data governance processes remain responsive, inclusive, and democratically accountable (67, 68).

The work involved in maintaining sovereignty only makes sense when the controlled resource has some type of value and can be sustainably used.^2^ This requires a mechanism to (a) develop and maintain quality control measures to avoid health data pools being infested with data that has been fabricated or falsified, (b) make sure privacy and confidentiality is protected to maintain public trust, and (c) establish protocols to facilitate data reuse and reproducibility (69).

To facilitate control over health data, it has become common among ethicists to suggest the principle of data minimalism or data minimisation to avoid the collection of unnecessary data and thereby reduce chances of extortion, discrimination, and other unethical data practices, as well as reduce the work required to monitor how data is being used (37, 70). Furthermore, particularly in the European policy context, it has been acknowledged that to maintain sovereignty over digital infrastructures, unilateral dependencies on data processors outside of one's jurisdiction need to be minimised and avoided to reduce unwanted uses of data or vulnerability (4, 71–73). In this sense, health data sovereignty is dependent on digital sovereignty (74). This requires establishing policies to reduce levels of technological dependencies (75) and develop local know-how to increase control over digital infrastructure, which is a precondition for data sovereignty.

### 3.4 Sovereignty for the common good

To maintain sovereignty, it is crucial to *protect the reasonable interests of all community members*. Efforts to achieve sovereignty have to aim at advancing control rights for all its community members and not merely securing privileges for some members at the expense of others. In particular, sovereign rights should not lead to a tyranny of the majority, as this would lead to a situation where an increasing number of members of a community or identity group will not recognise themselves as sovereign. Maintaining sovereignty within a group does not allow the disenfranchisement or systematic neglect of its members. At the same time, equitable protection of interests sometimes requires differential treatment. To honour the above-mentioned subgroup specific interests and respond to special vulnerabilities, communities may have to establish special mechanisms to secure interests such as privacy and non-discrimination. This means, for example, that community members – especially those structurally disadvantaged – need to be informed and provided sufficient access to information about how their data are being used to be able to contextualise and correct inaccurate or harmful representations (76).

While harm prevention is a key interest, sovereign societies need to also acknowledge the positive potential of health data. Sovereign control of health data can advance social well-being, particularly by improving healthcare and public health, and allow the development of new tools and marketable products. A recent example of this is the global, patient-led initiative “#WeAreNotWaiting community” formed by people with diabetes to develop open-source automation algorithms for insulin delivery outside of clinical settings (77, 78). Sharing data within the community was common to improve the system and integrate it into more formal research practices (79). A similar project, but without addressing one specific health condition, is the community-based online platform “Open Humans,” which enables users to collect, share, authorise access to, and control their data, thereby supporting both global academic research and patient-led projects (80). The success of such efforts not only depends on retaining control over data, especially by avoiding signing exclusive licences, but also on improving access to data. This familiarity and adherence to open science practices, such as FAIR principles (findable, accessible, interoperable and reusable) (81), are even more pressing to advance the common good.

### 3.5 Valuing sovereignty and solidarity

While self-determination is a central ethical principle in the health sector, there are other ethical principles that need to be considered when it comes to maintaining or ceding control of health data. As some health data are more valuable than other, for instance, during the early stages of the outbreak of a new pathogen, or of other exceptional interests for health research (82, 83), we need to ask ourselves about the relationship between *health data sovereignty and solidarity*, particularly to those outside one's community and identity group. The widely accepted principle of benefit-sharing would make it ethically acceptable to make sharing conditional on having reciprocal access to any products, data or knowledge based on research on or with the shared good. Yet when nothing of interest can be provided in exchange, there might be the need to share health data altruistically, as a matter of solidarity (84), to do our part to promote the health equity and well-being beyond our communities (85, 86). The practice of voluntarily sharing personal data, without necessarily having direct benefit, is also described as data donation (87) and is particularly important for solidarity-based healthcare systems to monitor the effectiveness of health interventions (88). Collective sovereignty makes it likely that people will also consider their reputation among neighbours and act on humanitarian grounds. It may also build up sufficient commitment to participate in international scientific cooperation by sharing health data as a matter of solidarity and to be in a better position to face health threats (89). Some societies may embrace solidarity as a central ethical value that ought to be promoted and defended while exercising sovereign rights. Moreover, where health data sharing practices were traditionally motivated by a solidaristic commitment towards the common good, the commercialisation of these data may disrupt willingness to continue sharing data when benefit-sharing terms remain unclear or are perceived as unfair (90, 91). Tensions and a reckless pursuit of community-based short-term interests may imperil health data sharing practices, even if this poses a severe health risk to others. At the same time, there must be no negative repercussions for communities that share health data for the common good, particularly when tackling global health threats (92).

### 3.6 Coherence and tensions across the five dimensions

The five dimensions set out above are not independent observations about the sovereignty elements of HDS but interrelated conditions where joint satisfaction defines what we take legitimate HDS to require. They also stand in tension with one another at several points. Making these tensions explicit, and indicating how we weighed them in our analysis, is necessary both to ensure methodological transparency and to clarify the action-guiding implications of our account.

A first tension arises between the pluralistic distribution of legitimate claims (3.1) and the collective-maintenance demands of sovereignty as an aggregate public good (3.2). Recognising that individuals, genetic relatives, healthcare workers, curators, researchers, and the state may all hold defensible claims risks fragmenting sovereignty to the point where no party can sustain it. Conversely, treating sovereignty as a collective good that depends on disciplined participation risks subordinating individual claims to majoritarian preferences. We resolved this tension by treating pluralism as a constraint on how collective sovereignty is exercised rather than as a competing object of governance. Collective sovereignty is legitimate only where the plural structure of claims is recognised in deliberation and institutional design; collective discipline does not override individual claims but is the condition under which individual claims can be effectively defended against dominant actors with incompatible interests.

A second tension arises between the required individual commitment towards collective aims (3.2) and the protection of reasonable interests of all members (3.4). Maintaining sovereignty may require some coercion to respect rules agreed by a majority, but such coercion can readily slide into majoritarian domination of structurally disadvantaged subgroups. We treat non-domination as having priority in cases of conflict: where the maintenance of collective sovereignty would require sacrificing the reasonable interests of vulnerable subgroups, the appropriate response is institutional redesign rather than to sacrifice individual interests. This priority follows from the observation that HDS that dominates its own members is self-undermining, since affected members will not recognise themselves as sovereigns.

A third tension arises between minimising maintenance costs (3.3) and the additional work required to ensure health data resources can be put in use for solidarity efforts (3.5). The institutional, infrastructural, and epistemic conditions for maintaining sovereignty are demanding, and the temptation to conserve resources by limiting outward-facing commitments is real. We hold that solidarity, while genuinely in tension with closure, is not morally optional: communities that achieve sovereignty through what outsiders have shared for the development of digital and epistemic infrastructures inherit ethical obligations to reciprocate outside their borders, particularly when health data of substantial value for shared health threats is at stake. Solidarity is therefore not a residual virtue exercised after sovereignty is secured, but a constitutive element of the legitimate exercise of HDS.

Taken together, the five dimensions function less as parallel concerns than as a structured account in which (3.1) specifies who may claim sovereignty, (3.2) and (3.3) specify what its exercise requires, and (3.4) and (3.5) specify the constraints under which it remains legitimate.

## 4 Discussion

### 4.1 The five dimensions in the context of the European health data space regulation

The EHDS seeks to balance individual interests and social needs while retaining the capacity to defend itself from unwanted outside interference, an aim that has substantial overlap with efforts to achieve sovereignty over health data. In Table 1 we provide a preliminary overview of how our five dimensions in realising HDS are targeted by different recitals and provisions of the Regulation 2025/327.

**Table 1:** Examples of overlaps between the five dimensions of HDS and targets of the regulation (EU) 2025/327 on the EHDS.

Dimension	Key element	Mapping to regulation (EU) 2025/327	Examples of relevant recitals (r) and provisions (p)
Build consensus	Agreement on roles	Definition of roles and responsibilities of health data holders, users, and access bodies	Specification of roles [r.79]; role of the Commission [p.96]; designation of contact point for secondary use [p.75]; function of the EHDS board [p.92, 94]
	Improve participation	Encourages involvement of citizens and stakeholders	Establishment of stakeholder forum [r. 97, p.93], improving data literacy [r.89]
Acknowledge public goods character	Health data as a public good	Recognises societal value of health data for research and public health	Overriding opt-out in emergencies [r.18]
	Importance of cooperation	Need to cooperate for control, useability and public benefit	Cooperation [r.30,32,57,64,65,87,95,99, p.19,55,57]
Reliably maintain control	Ongoing data protection	Control over access and data minimisation	Control role [r.72,77,79, p.74]; data minimisation [r.3, 19,72, p.66,68]; security [r.4,29,30,36,43,46,49,60,77; p.28,36,44,55,68,73,87]
	Maintain social value of health data	Establish interoperability standards to ensure that health data remains a valuable resource	Interoperability [r.19, 25,26,33,49,80,105, p.15,47,48]; standardisation [r.25,30:95]; acknowledgement of social value [r.58,85]
	Individual control mechanisms	Empowerment to control	Training and literacy [r.89]; opt-out mechanisms [r.54, p.10,71]
Honour all members’ interests	Provide room for different voices to express concerns and needs	Balances needs and concerns of patients, professionals, researchers, industry	Establishment of stakeholder forum [r. 97, p.93]
	Establish safeguards	Prevent misuse of health data and establish measures for ensuring anonymity	Prevention of misuse [p.67]; data permits [p.68]; data requests [p.69]
	Empower all groups	Establish data literacy programs specially tailored to the needs of diverse groups	Improving data literacy [r.89]; facilitate accessibility for all [p.4]
Balance values	Alignment with community values	Embedment of core values, such as confidentiality, respect for autonomy, trust, altruism, privacy, and transparency	Confidentiality [r. 4, 24, 34, 46, 55, 93]; respect for autonomy [r.54]; trust [r.4]; altruism [r.78]; privacy [r.65,72,77,80,93]; transparency [r. 16, 48, 49, 66, 68, 69, 70, 85]
	Long-term commitment to remain in line with community values	Secondary use limited to public interest purposes (research, policy, innovation) and maintain fair access	Fair data principles [r.3], fair prices [r. 61]; refrain from action incompatible with duties [r.64]; principle of proportionality [r.93]
	Balance social needs with respect for autonomy	Acknowledges the public benefit of exhaustive and representative data sets while recognising the importance of respecting autonomy	On balancing interests [r.54]

While this overview fulfils mostly illustrative purposes, it allows us to discuss three particularities. First, while our analysis on HDS singled out solidarity as a key social value, the Regulation explicitly highlights a much broader spectrum of values, such as privacy and confidentiality, security, transparency, trust, altruism and autonomy. Yet, even though the EHDS emphasises privacy and security, experts still demand improvements in relation to cybersecurity (93). Second, the Regulation places a much stronger emphasis on society benefiting from health data than we did in our analysis of HDS, specifying conditions for efficient use and cooperation (e.g., interoperability) by both the public and private sectors. This is a welcomed step, although critics warn that high financial and administrative cost may limit the ability of start-up and medium-sized companies to benefit from and contribute to these measures, including smaller and medium-sized hospitals and research organisations (94). Third, while the Regulation recognises the need to empower individuals, a significant omission is the lack of recognition of group specific risks and the need to support capacity-building at the group level so that communities with special needs are empowered to defend their interests and bridge digital divides. This is a point our analysis highlights on the basis of scholarship in the area of Indigenous data sovereignty (5). In this regard, first commentaries on implementing the EHDS Regulation already point to substantial inequalities within European countries with regard to benefiting from the EHDS (95). To reduce inequalities and improve social acceptance, member States and the EU need to establish major initiatives to develop communication strategies that recognise the population diversity and to expand training programs and capacity-building (96–99).

### 4.2 Future steps towards health data sovereignty

The nature of our conceptual-ethical analysis allows us to only suggest general lines of work for practical implementation. On the basis of the five dimensions we have examined, for societies that embrace HDS as a socially desirable goal we recommend three lines of work: (a) facilitate the material conditions that make it easier for people to come together and exert sovereign control, (b) develop civic education campaigns to resolve ambiguities over what it is to be sovereign over health data, and (c) offer actionable guidelines on how to build and maintain sovereignty.

For the first task, governments, international organisations, and civil society organisations need to work towards ensuring that digital infrastructures allow democratic control and that people have both the physical space and freedom to come together for collective action. Current political and economic developments have shown that the digital infrastructure we depend on is increasingly outside democratic control and vulnerable to the reckless advancement of private and external interests at the cost of the common good (24, 73, 100, 101). Alternative technologies and tools need to be offered. Furthermore, community-building can and should be stimulated by making it easier for people to meet and discuss their concerns over health data uses. This requires making common spaces such as city parks and community buildings accessible for citizen initiatives.

Concerning the second task, civic education campaigns need to increase the understanding of the general public and policymakers on the work, knowledge and skills required to achieve and maintain control over data (102). Ideally such efforts need to use an extensive and diverse set of didactic tools to maximise the extent of the audience that they can reach and have to be designed to avoid exacerbating the digital divide by paying attention to potential barriers in learning processes, such as language competency and accessibility.

Finally, as a third task, academia, international organisations and governmental civic education offices need to offer actionable guidelines on how to build and maintain sovereignty, including how to foster continuous commitments, address power asymmetries and provide room for community values, such as solidarity.

## Data Availability

The original contributions presented in the study are included in the article/Supplementary Material, further inquiries can be directed to the corresponding author.

## Footnotes

^1^This is a key distinction between data sovereignty and other concepts to align uses of data with the interests of data subjects. For instance, data trusts foresee that control functions are assumed by specialists who have the duty to act in the data subjects’ interests, see (38, 62).

^2^The related concept of data stewardship is particularly strong in emphasising the different responsibilities that need to be assumed to sustainably manage data and conserve its value as scientific resource. The multiple understandings of data stewardship do not allow us to draw systematic comparisons with HDS within the scope of this article. See, for example (69) for a concise overview of the heterogeneity of the concept.
